# Molecular regulation of myocardial proliferation and regeneration

**DOI:** 10.1186/s13619-021-00075-7

**Published:** 2021-04-06

**Authors:** Lixia Zheng, Jianyong Du, Zihao Wang, Qinchao Zhou, Xiaojun Zhu, Jing-Wei Xiong

**Affiliations:** Beijing Key Laboratory of Cardiometabolic Molecular Medicine, Institute of Molecular Medicine and State Key Laboratory of Natural and Biomimetic Drugs, Peking University, Beijing, 100871 China

**Keywords:** Cardiac regeneration, Cardiomyocyte proliferation, Signaling pathways, Zebrafish, Mouse

## Abstract

Heart regeneration is a fascinating and complex biological process. Decades of intensive studies have revealed a sophisticated molecular network regulating cardiac regeneration in the zebrafish and neonatal mouse heart. Here, we review both the classical and recent literature on the molecular and cellular mechanisms underlying heart regeneration, with a particular focus on how injury triggers the cell-cycle re-entry of quiescent cardiomyocytes to replenish their massive loss after myocardial infarction or ventricular resection. We highlight several important signaling pathways for cardiomyocyte proliferation and propose a working model of how these injury-induced signals promote cardiomyocyte proliferation. Thus, this concise review provides up-to-date research progresses on heart regeneration for investigators in the field of regeneration biology.

## Background

Myocardial infarction (MI) is a devastating disease worldwide. At present, nearly 40 million patients are suffering from heart failure. Due to the lack of an adequate blood supply, ~ 25% of total cardiomyocytes (CMs) undergo apoptosis and necrosis within a few hours after MI (Murry et al. 2006). Since the regenerative capacity is poor, this massive loss of CMs eventually leads to cardiac fibrosis and heart failure. The main clinical therapies for heart failure include drug intervention, left ventricular assist device implantation, and heart transplantation (Heallen et al. 2019). However, current drug and assist device therapies cannot reverse the CM loss while donor hearts for transplantation are limited. It remains challenging to find effective therapeutic methods for heart failure. Over the past decades, cardiac regeneration has become one of the most exciting research topics in the search for innovative interventions and therapies for heart failure, consisting of induced pluripotent stem cell- and embryonic stem cell-derived CM transplantation, cardiac tissue engineering, stimulating endogenous CM proliferation, and reprogramming non-myocytes into CMs (Tzahor and Poss 2017; Sadek and Olson 2020).

After two decades of debate, it is well accepted that the so-called adult cardiac stem cells (CSCs), including c-Kit^+^ CSCs, make no or minimal contributions to cardiac repair, while recent studies do not support the existence of adult CSCs (Li et al., 2018a; Sultana et al. 2015; van Berlo et al. 2014). Besides, many investigators have paid attention to studying regenerative environments or niches, such as cardiac fibrosis, inflammation, and coronary vessel regeneration (Zeisberg and Kalluri 2010; Swirski and Nahrendorf 2013; Bardot and Dubois 2019; Kramann et al. 2015; Bajpai et al. 2018). Some lower vertebrates, such as zebrafish and axolotl, have a strong regenerative ability in the adult heart and other organs (Poss et al. 2002; Cano-Martinez et al. 2010). But in mammals, efficient cardiac regeneration only occurs in newborns, and this ability is lost a few days after birth. Adult mammalian CMs renew at a very low rate (Kajstura et al. 2010; Bergmann et al. 2009), and newly-generated CMs are likely derived from existing CMs (Ali et al. 2014; Senyo et al. 2013). Re-activating the regenerative potential in adult CMs may be a new alternative to replenish the lost CMs after MI. There are some excellent review articles regarding CM transplantation, cardiac tissue engineering, and reprogramming fibroblasts into CMs (Laflamme and Murry 2011; Vunjak-Novakovic et al. 2011; Srivastava and DeWitt 2016; Keepers et al. 2020). In this brief review, we focus on the current understanding of the molecular and cellular mechanisms underlying CM proliferation and heart regeneration.

## Heart regeneration in model organisms

Regenerative capacity is quite different among organs and species. Some invertebrates, such as *Hydra* and planarians, can regenerate the whole body (Tanaka and Reddien 2011; Duncan and Sanchez, 2019). It was initially reported that ventricular injury induces CM proliferation but forms cardiac fibrosis in the adult newt, and later confirmed by others to support cardiac regeneration with minimal fibrosis (Oberpriller and Oberpriller 1971; Oberpriller and Oberpriller 1974; Witman et al. 2011; Mercer et al. 2013). The zebrafish has become a well-recognized model organism for studying embryonic development, organ function, and behavior, as well as organ regeneration including the heart (Poss et al. 2002; Raya et al. 2003). The zebrafish heart can fully regenerate after up to 20% ventricular resection (Poss et al. 2002). Others have reported a cryoinjury model of adult zebrafish heart that better mimics MI in mammals including human beings (Chablais et al. 2011; González-Rosa et al. 2011; Schnabel et al. 2011). In addition, genetic ablation of CMs in the adult zebrafish heart leads to an inflammatory response, endocardial and epicardial activation, and myocardial proliferation that is similar to the resection and cryoinjury models (Wang et al. 2011).

Although the adult mammalian heart has limited regenerative capacity, the mouse heart has full capacity to regenerate after ventricular resection at postnatal days 1-6 (P1-6), but loses this capacity by P7 (Porrello et al., 2011a). Others have argued that regeneration of the neonatal mouse heart is limited after apical resection, where the regenerative capacity is dependent on the surgical method, the size of the resection, and the evaluation method (Bryant et al. 2015; Andersen et al. 2016). Like the zebrafish heart, genetic ablation of CMs in the fetal mouse heart triggers a proliferative response in CMs and restores the morphology of the injured heart (Sturzu et al. 2015). In the cryoinjury model, the regenerative capacity of the neonatal mouse heart is dependent on the severity of injury. The neonatal heart can fully regenerate after non-transmural cryoinjury, but not after transmural cryoinjury which results in more CM loss and more severe fibrosis (Darehzereshki et al. 2015). Unlike other types of injury models, cryoinjury does not trigger significant numbers of proliferating CMs but frequently causes angiogenesis and epicardium activation (Darehzereshki et al. 2015; Malek et al. 2017). The neonatal mouse heart can also fully regenerate after left anterior descending coronary artery ligation (Mahmoud et al. 2014; Blom et al. 2016; Sereti et al. 2018). Also, the regenerative response of the neonatal heart has been demonstrated in large mammals such as pigs, with full regeneration without fibrosis at P1 and P2, and gradual loss of regenerative capacity from P3 to P14 (Zhu et al., 2018a; Ye et al. 2018). Even the neonatal human heart can recover from MI (Haubner et al. 2016). The heart regeneration models share the common feature of injury-induced CM proliferation and the resolution of fibrosis. Together, both the zebrafish and the neonatal mammalian heart have a full capacity to regenerate after different types of injury although the injury-induced responses are not identical. Compared with CMs that are terminally differentiated and quiescent in the adult zebrafish and newt heart, neonatal mouse CMs are immature and easily gain cell-cycle re-entry, thus it is better to consider the neonatal mouse heart as an injury-induced developmental model.

Myocardial regeneration capacity varies among species and changes dramatically within a week after birth in rodents. It is interesting to look at these differences from a developmental and evolutionary perspective. There are significant differences in heart structure, blood pressure, body temperature, oxygen content in the living environment, energy metabolism, and cardiac cell structure during individual development and evolution. From fish to mammals, the oxygen content of their living environment increases dramatically. This same change occurs right after birth in mammals. There is evidence that low-oxygen environments can stimulate regenerative potential (Kimura et al. 2015; Nakada et al. 2017). Hyperoxic environments may also affect the energy metabolism of CMs (Nakada et al. 2017). There are also significant differences in the structure of CM during evolution. Adult mammalian CMs have a more complete sarcomere structure. It has been shown that the CM proliferation is greatly related to the depolymerization of the sarcomeres (Ahuja et al. 2004). Others have found that the proportion of diploid CMs, which is thought to represent the regenerative capacity of CMs, is negatively correlated with metabolic rate in vertebrates, as well as body temperature in mammals. Also, they found that plasma thyroxine (T4) levels are inversely correlated with the proportion of diploid CMs. Inhibition of thyroid hormone signaling enhanced CM proliferation and regeneration (Hirose et al. 2019).

## Cellular mechanisms underlying heart regeneration

After ventricular resection in zebrafish, a clot forms rapidly, followed by fibrin and collagen deposition from 2 to 9 days post-amputation (dpa). During the course of regeneration, newly-formed myofibers gradually replace the collagen and the fibrin clot to achieve a fully functional heart by ~ 60 dpa (Poss et al. 2002). Lineage tracing has revealed that the lost CMs are replenished by pre-existing cardiomyocytes in the adult zebrafish heart (Jopling et al. 2010; Kikuchi et al. 2010). Ventricular CMs can be transdifferentiated and regenerated from atrial CMs after injury, suggesting that the embryonic heart has greater plasticity (Zhang et al. 2013). Using the Rainbow-based lineage mapping method, others have revealed that the cortical layer contributes to the formation of new CMs during heart development and regeneration (Gupta and Poss 2012). A Cre-LoxP-based lineage tracing study of tbx5a-expressing CMs has found that trabecular CMs can generate cortical CMs for myocardial regeneration after injury (Sanchez-Iranzo et al., 2018a). However, the molecular and cellular changes in CMs during heart regeneration remain incompletely understood. It is now conceivable that they undergo sarcomeric disassembly with disorganized actin and myosin filaments and larger intercellular spaces during regeneration (Jopling et al. 2010). Some embryonic factors such as *gata4* are re-expressed in CMs affected by injury (Jopling et al. 2010). The loss of Gata4 function blocks myocardial regeneration and is accompanied by severe scars (Gupta et al. 2013). In addition, a pool of undifferentiated CMs are formed in the injured, wound-healing tissues, and embryonic cardiac transcription factors and myosin heavy chain 7 are re-activated in these cells (Lepilina et al. 2006; Sallin et al. 2015). Another study has reported that the transcriptional profiling of CMs during regeneration is similar to that during heart development (Aguirre et al. 2014). Single-cell RNA-seq has confirmed that CMs in the border zone of the injured heart undergo de-differentiation and have transcriptional profiling similar to embryonic CMs (Honkoop et al. 2019). Together, heart regeneration in zebrafish naturally engages in CM de-differentiation and proliferation.

The regeneration process in the neonatal mouse heart is similar to that in zebrafish. After partial apical resection, a blood clot forms and later is replaced by newly-formed cardiac tissue in the injury site. Lineage tracing has shown that newly-formed CMs come from pre-existing CMs (Porrello et al., 2011a; Sereti et al. 2018; Porrello et al. 2013). Consistently, others have also demonstrated that non-CMs contribute to newly-formed CMs in developing embryos but not in the adult heart during homeostasis or after injury by using a dual genetic lineage tracing strategy (Li et al. 2019). Therefore, it is generally assumed that newly-formed CMs are from pre-existing CMs but not cardiac stem cells or cardiac progenitor cells in the adult heart. Like zebrafish, *Gata4* is expressed in CMs in the neonatal mouse heart, and loss of *Gata4* in CMs increases the scar size while its overexpression leads to improved regeneration in the neonatal heart after cryoinjury, probably through sarcomere disassembly and de-differentiation (Malek et al. 2017). Multicellular transcriptional analysis has shown that the neonatal heart retains a permissive embryonic developmental state, which may allow CMs to re-enter the cell cycle and continue to proliferate after injury (Quaife-Ryan et al. 2017). Single-nucleus RNA-seq analysis suggests that a sub-group of CMs preferentially enters the cell cycle and mitosis after injury (Cui et al. 2020). Together, new CMs are regenerated from CM de-differentiation and proliferation in the zebrafish and neonatal mouse heart.

The difference in cardiac regeneration across species and age groups may be related to the degree of CM polyploidization. Fetal or neonatal CMs are mostly mononuclear with a diploid genome in mammals and zebrafish. After birth, DNA can replicate, however, the ability of karyokinesis or cytokinesis varies between species. In rat CMs, after birth, DNA replicates but with no cytokinesis, increases binucleated CMs from day 4, and reaching 90% on day 12 (Li et al. 1996). In humans, CM DNA replicates without karyokinesis or cytokinesis, resulting in mononuclear CMs with tetraploid or higher DNA content (Laflamme and Murry 2011). The proportion of polyploid CMs in human hearts increased significantly from age 10 to 20 years (Mollova et al. 2013). Unlike mammals, zebrafish CMs do not undergo polyploidization, and more than 95% of CMs are mononuclear and diploid. There is evidence that diploid CMs have greater regenerative potential. Consistently, genetically modified zebrafish hearts containing 50% of polyploid CMs fail to regenerate after injury (Gonzalez-Rosa et al. 2018). Tnni3k knockout mice show more mononuclear diploid CMs and improved CM proliferation while Tnni3k overexpression in zebrafish leads to implicated cardiac regeneration (Patterson et al. 2017).

Besides CM proliferation after injury, other cardiac cells are also activated to participate in regeneration (Table 1). In zebrafish, cardiac injury stimulates the epicardium to promote myocardial proliferation, and the activated epicardium mainly forms epicardial cells, subepicardial cells, and perivascular cells, but rarely endothelial cells and CMs (Kikuchi et al., 2011a). The activated epicardium after injury also promotes angiogenesis and contributes to the formation of cardiac fibroblasts, which is necessary for cardiomyocyte proliferation (Gonzalez-Rosa et al. 2012; Sanchez-Iranzo et al., 2018b). During neonatal mouse heart regeneration, the epicardium is also activated after injury (Porrello et al., 2011a). Although embryonic epicardial cells are pluripotent, investigators have revealed that *Tbx18*^*+*^ epicardial cells only give rise to minimal numbers of CMs in the neonatal heart after injury (Cai et al. 2019). Adult epicardial cells are unable to produce CMs, but regulate cardiac regeneration via paracrine effects (Zhou et al. 2011). Similarly, the endocardium is activated within hours post-injury in zebrafish. *Raldh2*, the enzyme for synthesizing retinoid acids, is quickly induced in the endocardium and epicardium, and retinoic acid signaling is essential for heart regeneration (Kikuchi et al., 2011b).

**Table 1:** Cellular responses upon cardiac injury

	Zebrafish	Neonatal mammals	Adult mammals
Endothelial cells (ECs)	Raldh2 increased in endocardium and retinoic acid signaling from endocardium is required for CM proliferation (Kikuchi et al., 2011b). Revascularization occurs via angiogenesis (Marin-Juez et al. 2016).	Arterial ECs migrate to the infarcted region and form collateral arteries in mice (Das et al. 2019).	Preexisting endothelial cells form new coronary blood vessels after injury in mice (He et al. 2017). Endocardial contribution to coronary arteries or vascular ECs after injuries is very limited (Tang et al. 2018).
CMs	Most of the adult CMs are mononuclear diploid cells and retain robust proliferative capacities (Poss et al. 2002; Gonzalez-Rosa et al. 2018). Regenerated CMs derive from the proliferation of preexisting CMs (Jopling et al. 2010; Kikuchi et al. 2010)	Cardiomyocytes are mononuclear diploids. After injury, pre-existing CMs are the source of cardiac regeneration (Porrello et al., 2011a).	Most adult CMs are polyploid. The level of CM proliferation after injury is very low (Bergmann et al. 2009; Ali et al. 2014).
Fibroblasts	Fibroblasts synthesize ECM collagen post-injury and are inactivated during scar resolution. Both fibroblasts and the ECM are necessary to stimulate CM proliferation and regeneration (Sanchez-Iranzo et al., 2018b).	ECM deposit after injury but only minimal fibrotic tissue can be observed 21 days after injury (Porrello et al., 2011a).	Fibroblasts proliferate, and deposit ECM after myocardial infarction.
Epicardium	Epicardium is activated after injury and restricted to injury area 7 days after injury. Epicardial derived cells differentiate into perivascular cells and myofibroblasts, but not CMs, or coronary endothelium (Kikuchi et al., 2011a; Gonzalez-Rosa et al. 2012).	Epicardium is activated after injury with increased expression of Wt1 and Raldh2. Tbx18^+^ epicardial cells give rise to minimal numbers of CMs in the neonatal mouse heart after injury (Cai et al. 2019)	Epicardium-derived cells do not produce cardiomyocytes, but secrete paracrine factors to regulate heart regeneration (Zhou et al. 2011)
Macrophages	Depletion of macrophages leads to impaired heart regeneration (Lai et al. 2017).	Embryonic-derived resident cardiac macrophages increase after injury and are essential for cardiac repair (Lavine et al. 2014).	Monocyte-derived macrophages were recruited after injury. Inhibition of Monocyte recruitment improves cardiac repair (Lavine et al. 2014).

**Table 2:** Major signaling pathways regulating heart regeneration

**Mammalian pathways**	**Molecules**	**Experiments**	**Functions in CM Proliferation**	**References**
Cdk/cyclin complex	cyclin D2	OE in adult mice	Increases DNA synthesis of CMs	Pasumarthi et al. 2005
	cyclin A2	OE in mice	Enhances CM proliferation and left ventricular systolic function	Chaudhry et al. 2004
	cyclin B1:CDC2	OE in rat CMs	Re-initiates cell division of adult rat CMs	Bicknell et al. 2004
	cyclin G1	OE in neonatal mice	Increases CM DNA synthesis but inhibits cytokinesis	Liu et al. 2010
	CDK1, CCNB, CDK4, CCND	OE in CMs	Induces mouse, rat, and human CM proliferation. Improves cardiac function after MI in mice	Mohamed et al. 2018
CDK inhibitors	p21, p27, p57	Combined knockdown	Promotes adult CM proliferation via increasing expression of cyclins A and E	Di Stefano et al. 2011; Tane et al. 2014
Upstream regulators	E2F1, E2F2	OE in adult mice	Increases DNA synthesis; increases CM apoptosis caused by OE of E2F1	Agah et al. 1997; Ebelt et al. 2008
Hippo	MST, LATS	KO	Induces CMs to re-enter the cell cycle	Heallen et al. 2013
	Salv	Knockdown or KO	Improves systolic cardiac function after MI in mice	Leach et al. 2017; Heallen et al. 2011
	YAP	Activation of YAP1 in mouse	Extends regenerative window in neonatal mouse heart and improves cardiac function after injury	Lin et al. 2014; Xin et al. 2013
		Constitutive activation	Makes adult CMs re-enter cell cycle by switching chromatin to a fetal-like and proliferative state	Monroe et al. 2019
		Administration of Agrin after MI	Enhances CMS proliferation in mice by interfering with YAP-DGC/DAG1 interaction	Bassat et al. 2017; Morikawa et al. 2017
Neuregulin	Nrg1/ErbB4	Nrg1 injection; ErbB4 OE or CKO	Nrg1 injection and ErbB4 OE promote CM proliferation after MI; CKO of ErbB4 in CMs reduces CM proliferation	Bersell et al. 2009; Polizzotti et al. 2015
	ErbB2	CKO in CMs	Reduces CM proliferation in embryonic or neonatal mouse	D'Uva et al. 2015
		Activation	Improves cardiac function post-MI	
Notch	Notch1/Jagged1	Virus delivery	Induces neonatal mouse CM proliferation	Felician et al. 2014
Wnt	Wnt	Chemical inhibition	Promotes CM proliferation in infarcted border zone	Yang et al. 2017; Xie et al. 2020
MAPK	MKK3/6	OE in mice	Causes premature heart failure	Liao et al. 2001
	p38	Activation	Suppresses neonatal CM proliferation	Engel et al. 2005
		Attenuation of p38 activity	Causes cardiac hypertrophy and increases mitosis of CMs	Braz et al. 2003; Engel et al. 2005
	Dusp6	Disruption in neonatal heart	Increases CM proliferation	Maillet et al. 2008
TGFβ	Fstl1	Epicardial patch/ myocardial injection	Promotes CM proliferation and improves cardiac function in mouse and pig heart	Wei et al. 2015
Epigenetic regulators	miR-15 family	Knockdown in neonatal mice	Increases number of mitotic CMs	Porrello et al., 2011b
		Inhibition in adult mice	Reduces infarcted area after ischemia-reperfusion	Hullinger et al. 2012
	miR199a	AAV9-mediated OE	Promotes neonatal and adult rat CM proliferation; promotes repair after MI in mice	Eulalio et al. 2012
		OE in pigs	Improves cardiac repair after MI, but most treated pigs died suddenly due to arrhythmia	Gabisonia et al. 2019
	miR302-367	OE in adult mouse	Promotes CM proliferation by regulating MST and LATS	Tian et al. 2015
	CPR	CKO in mouse CMs	Increases CM proliferation and improves cardiac function	Ponnusamy et al. 2019
	cirNfix	Knockdown	Promotes CM proliferation	Huang et al. 2019
	Baf60c	Knockdown	Suppresses CM proliferation in neonatal mouse heart	Nakamura et al. 2016
Transcription factors	Meis1	CKO in mouse CMs	Drives mature CMs to re-enter cell cycle	Mahmoud et al. 2013
		OE	Inhibits heart regeneration in neonatal mice	
	Gata4	Conditional KO	Leads to loss of regenerative ability in neonatal mice	Yu et al. 2016
	Tbx20	OE	Results in CM proliferation and improves cardiac function	Xiang et al. 2016
	REST	CKO in CMs	Inhibits CM proliferation in embryonic or adult mouse	Zhang et al. 2017
**Zebrafish pathways**	**Molecules**	**Experiments**	**Functions in CM proliferation**	**References**
Neuregulin	Nrg1	OE	Increases CM proliferation	Gemberling et al. 2015
	ErbB2	Inhibition	Reduces CM proliferation	Gemberling et al. 2015
	Vitamin D	Administration	Promotes CM proliferation dependent on Nrg1/ErbB2	Han et al. 2019
Notch	Mastermind-like	Conditional inhibition in ECs	Decreases CM proliferation	Zhao et al. 2019; Zhao et al. 2014
Wnt	Wnt	Chemical inhibition	Increases CM proliferation	Xie et al. 2020; Zhao et al. 2019
FGF	Fgfr1	OE of dn-Fgfr1	Blocks epicardial EMT, disrupting coronary neovascularization and arresting regeneration	Lepilina et al., 2006
PDGF	PDGF	Chemical inhibition	Reduces CM DNA synthesis and inhibits heart regeneration	Lien et al. 2006
MAPK	MKK6	OE	Impairs cardiogenesis and heart regeneration	Jopling et al. 2012
	p38α	Inactivation	Prerequisite to re-enter cell cycle	Jopling et al. 2012
	Dusp6	OE	Impairs heart regeneration	Han et al. 2014
		Deletion	Increases CM proliferation and decreases cardiac fibrosis	Missinato et al. 2018
	MEK1/2	Chemical inhibition	Down-regulates pERK and decreases CM proliferation	Liu and Zhong, 2017
		OE of dn-MEK1	Prevents angiogenesis and cardiac regeneration	
TGFβ	TGFβ type I receptors	Chemical inhibition	Impairs heart regeneration by attenuating CM proliferation and enhancing scar formation	Chablais and Jazwinska, 2012; Choi et al. 2013
	Myostatin, inhbaa	Myostatin OE; inhbaa KO	Results in decreased CM proliferation	Dogra et al. 2017
		Myostatin KO; inhbaa OE	Causes hyperplasia and hypertrabeculation with late stage pericardial edema	
VEGF	vegfa	OE in adult CMs	Leads to CM hyperplasia in absence of injury; impairs cardiac repair after injury	Karra et al. 2018
Jak/Stat3	Stat3	OE dn-Stat3 in CMs	Blocks regeneration in part by regulating Rln3a	Fang et al. 2013
Epigenetic	Brg1	Dn-Brg1 OE	Regulates CM proliferation by interacting with Dnmt3ab to increase methylation of cdkn1c promoter	Xiao et al. 2016
	H3K27me3	Conditional OE of H3.3K27M	Decreases K27me3 level and inhibits cardiac regeneration	Ben-Yair et al. 2019
	miR-101a	Depletion	Promotes CM proliferation at 3 dpa, but sustained inhibition of its expression increases fibrosis	Beauchemin et al. 2015
	miR-133	OE	Restricts CM proliferation and inhibits regeneration	Yin et al. 2012
		Depletion	Enhances CM proliferation	
	miR-99/100	OE	Leads to deficiency in zebrafish heart regeneration	Aguirre et al. 2014
Transcription factors	Gata4	dn-Gata4 OE in CMs	Impairs proliferation and heart regeneration	Gupta et al. 2013
	Hand2	OE in CMs	Increases CM proliferation after injury	Schindler et al. 2014
	NF-κB	OE of dn-IκBSR in CMs	Disassembly of sarcomeres, proliferation, and induction of gata4 regulatory sequences all disrupted after injury	Karra et al. 2015

Regeneration of coronary vessels is also critical for achieving heart regeneration. New vessels are formed in the injured area, and neovascularization provides oxygen and blood for the hypoxic infarcted region and is required for heart regeneration in zebrafish (Lepilina et al. 2006; Marin-Juez et al. 2016). As for the neonatal mouse heart, arterial endothelial cells migrate from capillaries to the infarcted region and form collateral arteries, which are essential for heart regeneration (Das et al. 2019). Besides, immune cells and nerve cells also contribute to zebrafish and neonatal mouse heart regeneration through complex interactions among different types of heart cells (Aurora et al. 2014; Han et al. 2015; Kikuchi 2020; Mahmoud et al. 2015; White et al. 2015; Lai et al. 2017). 

Recently, several important studies have shown that the lymphatic system is also essential for cardiac regeneration and repair. The main function of the lymphatic vessels is to drain lymph and transport inflammatory cells. The abnormal lymphatic function lead to myocardial disease, arrhythmias, atherosclerosis, and other diseases (Norman and Riley 2016). Klotz and colleagues find that vascular Endothelial Growth Factor C (VEGF-C) induce a stronger lymphangiogenic response and result in improved cardiac function after myocardial infarction (Klotz et al. 2015). The extracellular protein reelin (RELN) is recently identified as a lympho-endothelial secreted factor that enhances cardiac regeneration in neonatal mice and improves heart function after myocardial infarction (Liu et al. 2020). Extensive lymphangiogenesis is also observed in zebrafish hearts after cryoinjury. Lymphatic vessels are essential for zebrafish cardiac regeneration, and genetically modified fish lines with lymphatic defects fail to regenerate after cryoinjury (Harrison et al. 2019; Gancz et al. 2019). Together, various types of cells in the injured heart coordinate and interact to achieve precise tissue regeneration, but many of these intercellular mechanisms are not well understood (Table 1).

## Molecular mechanisms regulating cardiomyocyte proliferation

It is now well-recognized that injury-induced CM proliferation is the major cellular mechanism for heart regeneration in the zebrafish and neonatal mouse. Intensive studies have shown that a number of signaling pathways are involved in this complex process, in which the epicardium, endocardium/endothelium, fibroblasts, and leukocytes actively interact with CMs. We thus provide below a brief review of several essential signaling pathways that regulate CM proliferation.

### Cell cycle regulators

Mammalian CMs are generally considered to be in cell-cycle arrest, often accompanied by a stronger expression of cell-cycle inhibitors (p21, p27, and Rb) and weaker expression of cell-cycle activators (cyclins and CDKs) (Tamamori-Adachi et al. 2008; Sdek et al. 2011; Tane et al. 2014). Cell-cycle regulatory proteins show dynamic expression patterns during rat and mouse cardiac development. For example, cyclin A2, cyclin B1, CDK2, and CDK6 are significantly down-regulated in the heart at 7 weeks compared to embryonic day 18 (E18) (Liu et al. 2010). In the mouse heart, the expression levels of cyclins (cyclins A, B1, D1, and E) and CDKs (CDKs 1, 2, and 4) decrease from E12 or E16 until P0, and then increase with a peak around P5 (Ikenishi et al. 2012). CDK inhibitors such as p21, p27, and p57 are important negative regulators of the cell cycle for CMs. The expression levels of p21 and p27 increase during the development of both the rat and human heart. p57 is only expressed in the rat heart from E12 to E15, but remains detectable from the embryonic to the adult human heart (Ikenishi et al. 2012). The pocket proteins (Rb, p107, and p130) are expressed in CMs with quite different dynamics. For example, the expression level of Rb increases in the heart during late gestation and postnatal stages, and it plays a vital role in the cell-cycle exit of CMs (MacLellan et al. 2005).

To promote adult CM cell-cycle re-entry, investigators have attempted to carry out ectopic expression of cell-cycle regulatory genes in CMs in vitro and the mouse heart in vivo. Transgenic overexpression of cyclin D2 results in increased DNA synthesis by CMs in the adult mouse heart, but overexpression of other cyclin D subfamily members, such as cyclins D1 and D3, appears to have little or no effect on the cell-cycle progression of CMs (Pasumarthi et al. 2005). A series of studies has shown that forced expression of cyclin A2 results in enhanced left ventricular systolic function both in mouse and pig (Chaudhry et al. 2004; Cheng et al. 2007; Shapiro et al. 2014; Woo et al. 2006). The cyclin B1-CDC2 complex is the key regulator of the G2/M-phase transition in CMs, and its overexpression re-initiates cell division of adult cardiomyocytes in vitro (Bicknell et al. 2004). Combined knockdown of CDK inhibitors (p21 and p27) is also able to promote adult CM proliferation with evident cytokinesis via increasing the expression of cyclins A and E (Tane et al. 2014; Di Stefano et al. 2011). As the key mediators of cell-cycle progression, E2F transcription factors have been well studied to address their roles in regulating cell-cycle progression in CMs. Overexpression of E2F1 and E2F2 in adult CMs increases DNA synthesis, but leads to apoptosis (Agah et al. 1997; Ebelt et al. 2008). Recently, Srivastava and colleagues have identified four cell-cycle regulatory genes (CDK1, CCNB, CDK4, and CCND) that are sufficient to induce the proliferation of mouse, rat, and human CMs, and adenoviral-mediated expression of these genes significantly improves cardiac function after MI in mice (Mohamed et al. 2018). Although forced expression of some cell-cycle regulators stimulates DNA synthesis, most of them have little effect on inducing CM cytokinesis, and some even have inhibitory effects. For instance, forced expression of cyclin G1 increases DNA synthesis but inhibits cytokinesis in neonatal CMs (Liu et al. 2010). Besides, forced expression of cell-cycle regulators in CMs induces apoptosis in mice (Agah et al. 1997; von Harsdorf et al. 1999; Kirshenbaum et al. 1996). Therefore, the recent work suggests that the spatio-temporal dose-dependent overexpression of cell-cycle regulators is essential for the cell-cycle re-entry of CMs (Mohamed et al. 2018).

### Hippo signaling pathway

**Fig. 1:**
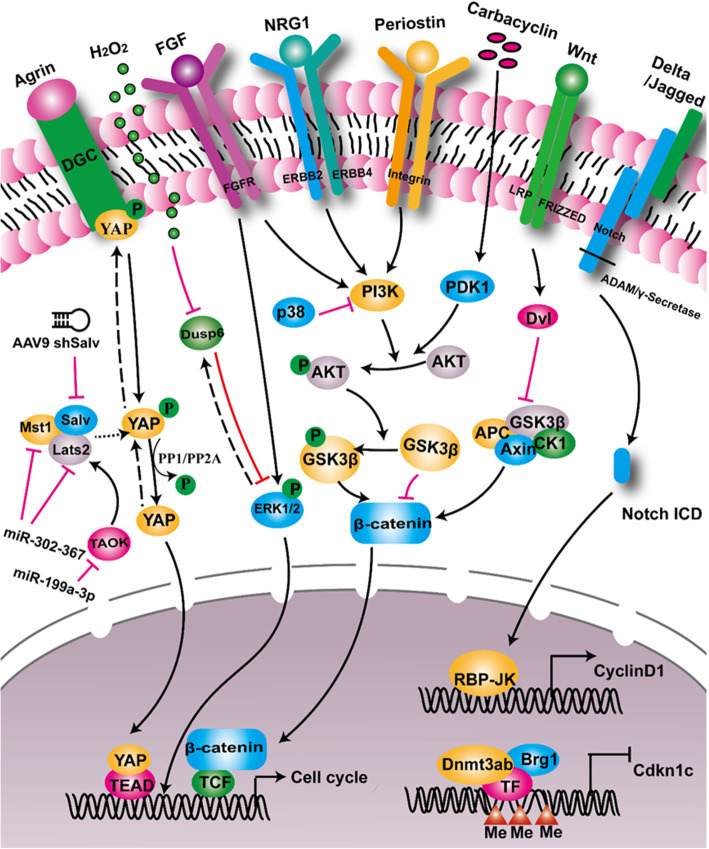
Summary of signaling pathways regulating cardiomyocyte proliferation. Hippo/YAP signaling is inactive when p-YAP is bound to the dystrophin glycoprotein complex (DGC). Binding of Agrin to the DGC leads to the translocation of p-YAP to the cytoplasm, phosphorylation of YAP is regulated by the kinase complex (Mst1/Lats2/Salv), and dephosphorylation of p-YAP by PP1/PP2A or others results in YAP activation and translocation to the nucleus. FGF signaling activates the MAPK pathway and induces the expression of Dusp6, a negative regulator of pERK, and Dusp6 protein is post-transcriptionally modified and degraded by H_2_O_2_. The PI3K-AKT signaling is regulated by NRG1/ErbB2/4, periostin/integrin, small molecule carbocyclin, and Wnt signaling pathways, which fine-tune β-catenin activity and its translocation into the nucleus. Binding of ligands such as Delta/Jagged to Notch receptors leads to Notch intracellular domain (NICD) activation and translocation to the nucleus. Together with injury-induced activation of chromatin remodeling factor Brg1/Dnmt3ab, the Yap/pERK-β-catenin/NICD activation in the nucleus regulates the expression of cell-cycle regulators including cyclins and cyclin inhibitors

Hippo signaling is a highly-conserved pathway that regulates organ size. It regulates embryonic heart development in both mouse and zebrafish (Fukui et al. 2014; Bornhorst et al. 2019; Artap et al. 2018). Its function in heart regeneration was first reported by Martin’s lab (Heallen et al. 2013). Some key components of this pathway, such as MST, LATS, and Salvador (Salv), regulate CM proliferation. Genetic deletion of either MST or LATS induces CMs to re-enter the cell cycle (Heallen et al. 2011). Either knockdown of Salv by shRNA or Salv knockout improves systolic cardiac function after MI in mice (Heallen et al. 2011; Leach et al. 2017)**.** YAP is a transcription coactivator and a downstream component of the Hippo pathway. Cardiac-specific knockout of YAP results in embryonic lethality (von Gise et al. 2012; Xin et al. 2011). Intriguingly, activation of YAP1 extends the regenerative window in the neonatal mouse heart beyond the first postnatal week, and improves cardiac function after injury, mostly by upregulating cell-cycle genes (Lin et al. 2014; Xin et al. 2013). PP1 and PP2A have been reported to activate YAP via de-phosphorylating p-YAP and translocating it into the nucleus (Haemmerle et al. 2017; Schlegelmilch et al. 2011). Administration of Agrin after MI leads to enhanced CM proliferation in mice (Bassat et al. 2017). Mechanistically, phosphorylation of YAP by Hippo enhances its interaction with dystroglycan 1 (DAG1), a component of the dystrophin glycoprotein complex (DGC), and thus inhibits the nuclear translocation of YAP (Morikawa et al. 2017). Agrin interferes with the YAP-DGC/DAG1 interaction, thus leading to YAP release, translocation into the nucleus, and cell-cycle progression by interaction with TEAD transcription factors (Bassat et al. 2017). A recent study further revealed that constitutive activation of YAP switches the chromatin to a more primitive, fetal-like, and proliferative state, making it easier for adult CMs to re-enter the cell cycle (Monroe et al. 2019). These studies suggest that inhibition of Hippo can be invoked as a strategy to promote CM regeneration for treating heart failure. However, others have found that Yap mostly regulates cell matrix and inflammatory gene networks and contributes to scar formation apart from CM proliferation during zebrafish heart regeneration (Flinn et al. 2019). In addition, Pitx2 has been identified as a transcription factor enriched in the regenerating Hippo-deficient mouse heart and is associated with the oxidative stress response. The Pitx2-deficient neonatal mouse heart fails to regenerate after apex resection, which probably affects mitochondrial function and adipose composition (Li et al., 2018b; Tao et al. 2016). Together, Hippo signaling negatively regulates CM proliferation via fine-tuning the YAP-DGC interaction, chromatin accessibility, and mitochondrial function (Fig. 1).

### Neuregulin/ErbB signaling pathway

Neuregulin1 (NRG1) is an EGF-like growth factor that binds with ErbB2 and ErbB4 receptors on the cell membrane of the CM. NRG1/ErbB signaling was first reported to promote CM proliferation and regeneration in mice, and was subsequently further shown to promote CM proliferation after MI or ventricular resection in the adult zebrafish and mice (Gemberling et al. 2015; Bersell et al. 2009; Polizzotti et al. 2015). During zebrafish heart regeneration, inhibition of ErbB receptors leads to a reduction of CM proliferation, while overexpression of Nrg1 increases it, suggesting that the Nrg1/ErbB signaling plays an important role in endogenous heart regeneration in zebrafish (Gemberling et al. 2015) (Fig. 1). Besides, others have found that the proliferative effect of vitamin D on CMs is dependent on Nrg1/Erbb2 signaling in zebrafish (Han et al. 2019). The conditional ablation of ERBB2 in embryonic or neonatal mouse CMs leads to reduced proliferation. Consistently, genetic activation of ERBB2 significantly improves cardiac function post-MI, indicating that this pathway can be exploited as a viable target for heart regeneration (D'Uva et al. 2015). To explore the potential therapeutic application of human recombinant NRG1 proteins (rNRG1), others have found that administration of rNRG1 immediately after cryoinjury is critical for promoting CM proliferation and regeneration using the P1 mouse heart, and rNRG1 has a similar effect of inducing proliferation in cultured CMs from pediatric patients (Polizzotti et al. 2015). Furthermore, a recent study has found that Nrg1/Erbb2 signaling induces metabolic reprogramming in the regenerating heart by switching oxidative phosphorylation to glycolysis in regenerating CMs (Honkoop et al. 2019). However, others have shown that rNRG does not induce CM DNA synthesis in either normal or infarcted adult mice (Reuter et al. 2014). Two clinical trials have shown dose-limited toxicity and a mild beneficial effect of rNRG1 in ~ 40 heart failure patients (Gao et al. 2010; Lenihan et al. 2016), therefore it remains to be determined whether rNRG1 has an effective clinical outcome for treating pediatric and/or adult heart failure patients.

### Notch signaling pathway

The Notch pathway plays a key role in the heart development of mammalian embryos. Mice lacking Notch receptors or ligands show early embryonic lethality mainly due to cardiovascular abnormalities (Penton et al. 2012). In addition to its function in embryonic heart development and cell lineage specification, Notch is also critical for CM proliferation (Collesi et al. 2008; Campa et al. 2008; Croquelois et al. 2008). During early postnatal development, Notch signaling is up-regulated to reactivate the cell cycle by inducing the expression and nuclear localization of Cyclin D1 (Campa et al. 2008). In zebrafish, the Notch pathway has been widely reported to promote CM proliferation (Raya et al. 2003; Zhang et al. 2013). Conditional inhibition of Notch signaling via the ectopic expression of Notch inhibitor dominant-negative mastermind-like in endothelial cells (including the endocardium) decreases CM proliferation (Zhao et al. 2019; Zhao et al. 2014) (Fig. 1). Others have shown that flow-responsive *klf2a* and *klf2b* are critical for regulating endocardial Notch signaling including increased *notch1b* expression after embryonic ventricular CM ablation (Galvez-Santisteban et al. 2019; Li et al. 2020), and endothelial Brg1 fine-tunes Notch signaling during adult zebrafish heart regeneration (Xiao and Hou et al., unpublished data). In addition, adeno-associated virus delivery of Notch1 or its ligand Jagged1 induces neonatal mouse CM proliferation, in which Notch activation increases the transcription of Notch1-targeted genes via establishing an open chromatin conformation (Felician et al. 2014). However, the effect of Notch signaling on the adult mouse heart after MI is very limited. DNA methylation analysis has shown that the promoters of Notch target genes are more highly methylated in the adult mouse CM than in the neonatal heart, thus leading to transcription repression (Felician et al. 2014). Together, these studies suggest an essential role of endothelial and endocardial Notch signaling in regulating myocardial proliferation and regeneration.

### Wnt signaling pathway

Wnt pathways include the classical Wnt/β-catenin pathway, the atypical planar cell polarity pathway, and the non-classical Wnt/Ca^2+^ pathway (Nusse and Varmus 2012). Wnt signaling plays vital roles in cardiac development, especially in myogenesis (Gessert and Kuhl 2010)**.** The classical Wnt/β-catenin pathway positively regulates cardiac specialization during the early stages of development, but plays an inhibitory role in the later stages. Wnt signaling also promotes the proliferation of progenitor cells in the second heart field of the mouse embryo (Bisson et al. 2015). In addition, both the classical and non-classical Wnt pathways play a role in injury-induced cardiac repair. The Wnt signaling pathways are relatively inactive in the adult mammalian heart, but are immediately activated upon injury (Hermans et al. 2012). Wnt ligands have various expression patterns during MI. Wnt1 is rapidly induced and persists for 2 days after MI, while Wnt4 and Wnt7a are transiently up-regulated in the later stages of MI (Duan et al. 2012)**.** These Wnt ligands activate both the classical and non-classical Wnt pathways. Others have recently reported that Wnt signaling regulates adult CM proliferation, and the small molecule CGX132146 and cardiomogen, inhibitors of Wnt signaling, promote CM proliferation in the infarcted border zone (Yang et al. 2017; Xie et al. 2020). Consistent with this, cardiac regeneration in mice with conditional knockout of Lrp6, a co-receptor of Wnt ligands, improves at least partly via promoting CM proliferation (Wu et al. 2020). In zebrafish, a transgenic reporter of Wnt/β-catenin signaling, TOPdGFP, is upregulated in the injured areas after ventricular resection (Stoick-Cooper et al. 2007), and inhibition of Wnt signaling increases CM proliferation (Zhao et al. 2019; Xie et al. 2020) (Fig. 1). Together, these studies suggest a biphasic action of Wnt signaling where Wnt ligands and receptors are induced upon injury but turning down Wnt signaling is essential for CM proliferation and regeneration after ventricular resection in zebrafish and MI in mice. This discrepancy warrants further mechanistic studies.

### MAPK signaling pathway

The MAPK signaling pathway is a classical kinase cascade pathway activated by growth factors such as fibroblast growth factor (FGF) and platelet-derived growth factor (PDGF), and it regulates cell proliferation, differentiation, and survival, as well as playing a key role in cardiovascular diseases (Muslin 2008). Both the FGF and PDGF signaling pathways are required for zebrafish heart regeneration by regulating the epicardial epithelium-mesenchyme transition, angiogenesis, and CM proliferation (Lepilina et al. 2006; Lien et al. 2006). In mice, MAPK signaling regulates CM proliferation during heart regeneration. MKK/p38α has been demonstrated to negatively regulate proliferation in both the zebrafish and mouse. In zebrafish, overexpression of MKK6 impairs cardiogenesis and heart regeneration, while inactivation of p38α is a prerequisite for re-entry into the cell cycle (Jopling et al. 2012). In mice, overexpression of MKK3 or MKK6 leads to premature heart failure (Liao et al. 2001). Besides, the activation of p38 suppresses neonatal CM proliferation, while attenuation of p38 activity causes cardiac hypertrophy and increases mitosis in CMs (Engel et al. 2005; Braz et al. 2003). Dual specificity phosphatase 6 (Dusp6), an ERK phosphatase, is a negative feedback factor in FGF signaling. Dusp6 is transiently induced after ventricular resection in zebrafish, and overexpression of *dusp6* impairs heart regeneration (Han et al. 2014), its deletion increases CM proliferation and angiogenesis, and decreases cardiac fibrosis after ventricular resection in zebrafish (Missinato et al. 2018). In the neonatal mouse heart, Dusp6 disruption leads to cardiac hypertrophy and increased CM proliferation (Maillet et al. 2008). In addition, MEK1/2 and ERK1/2 are upregulated after ventricular resection in zebrafish. Inhibition of MEK1/2 by ADZ6244 down-regulates pERK and thus decreases CM proliferation. Consistently, over-expression of dominant-negative MEK1K98M prevents Fli1^+^ endothelial cell migration into the injury site, angiogenesis, and cardiac regeneration (Liu and Zhong 2017). Thus, several components of the MAPK signaling pathway are responsive to injury and play essential roles in heart regeneration (Fig. 1).

### TGFβ signaling pathway

TGFβ signaling is an important pathway to regulate tissue fibrosis including cardiac fibrosis. In zebrafish, TGFβ ligands (Tgfb1, b2, and b3) and receptors (Alk4, Alk5a, and Alk5b) are induced after cryoinjury. Small-molecule inhibition of TGFβ type I receptors suppresses pSmad3 and impairs heart regeneration by attenuating CM proliferation and enhancing scar formation (Chablais and Jazwinska 2012; Choi et al. 2013). Fstl1, a TGFβ family member, has been shown to promote CM re-entry into the cell cycle. Delivering Fstl1 by epicardial patch or myocardial injection into the mouse and pig heart promotes CM proliferation and improves cardiac function. However, this pro-proliferative effect can only be achieved when Fstl1 is derived from epicardium but not from CMs (Wei et al. 2015). Similarly, during adult cardiac valve regeneration, TGFβ signaling promotes the re-entry of endothelial and hematopoietic cells into the cell cycle (Bensimon-Brito et al. 2020). These studies suggest that TGFβ signaling is essential for promoting endothelial and CM proliferation in the adult zebrafish and mouse heart after injury.

### Epigenetic regulation

Epigenetic regulation plays an important role in cardiac development and heart regeneration (Zhu et al., 2018b; Moore-Morris et al. 2018). It consists of multiple components, such as DNA methylation, histone modification (methylation, acetylation, and phosphorylation), chromatin remodeling complexes, and non-coding RNAs (microRNAs and long non-coding RNAs). During the past decade, major progress has been made, revealing the functions of microRNAs (miRNAs) in CM proliferation in the zebrafish and mouse. miRNAs are 21-23-base single-strand non-coding RNAs. The miR-15 family has been reported to regulate the cell cycle of CMs and cardiac remodeling in ischemic heart disease. A miR-15 family member, miR-195, is strongly induced in the P10 ventricle relative to P1, and knockdown of the miR-15 family in the neonatal mouse with locked nucleic-acid modified anti-miRNAs increases the number of mitotic CMs (Porrello et al., 2011b). Inhibition of the miR-15 family in the adult mouse and pig reduces the infarcted area after ischemia-reperfusion (Hullinger et al. 2012). Others have performed high-content fluorescence microscopy screening in neonatal rat CMs with a library of 875 miRNA mimics, and have identified a number of miRNAs including miR-199a, as important regulators of CM proliferation. AAV9-mediated overexpression of miR-199a promotes neonatal and adult rat CM proliferation as well as CM regeneration and repair after MI in mice (Eulalio et al. 2012). The same group has further demonstrated that over-expression of miR-199a improves cardiac repair after MI in pigs, but most of the treated pigs died suddenly due to arrhythmia (Gabisonia et al. 2019). Mechanistic studies have shown that miR-199a-3p directly binds to TAOK1, β-TRCP, and cofilin2 to inhibit Hippo signaling, thus leading to YAP activation and inhibition of actin depolymerization (Torrini et al. 2019). Similarly, overexpression of the miRNA cluster miR302-367 in the adult mouse heart down-regulates MST and LATS kinases in the Hippo signaling pathway, thus promoting CM proliferation (Tian et al. 2015). Recently, other microRNAs such as miR-19a/19b, miR-128, and miR-294 have also been reported to regulate CM proliferation in mice (Gao et al. 2019; Huang et al. 2018; Ponnusamy et al. 2019). Several miRNAs including miR-133, miR-101a, miR-99/100, let-7a/c, and miR-26a are critical for heart regeneration in zebrafish as reviewed previously (Zhu et al., 2018b).

In addition to microRNAs, other non-coding RNAs including long non-coding RNAs (lncRNAs) and circular RNAs (circRNAs) are also potential regulators of CM proliferation. Bioinformatics analysis has revealed that lncRNA CPR (Cardiomyocyte Proliferation Regulator) is a negative regulator of CM proliferation and regeneration (Ponnusamy et al. 2019). CircRNA is a special kind of noncoding RNA that has a closed circular structure. It is not affected by RNA exonucleases, and its expression is more stable. A recent study has revealed that cirNfix normally inhibits CM proliferation, and thus its knockdown promotes CM proliferation. Consistent with this, the super-enhancer region of cirNfix has binding sites for Meis1, a negative regulator of CM proliferation, and thus regulates the expression of cirNfix (Huang et al. 2019).

The SWI/SNF complex is an ATP-dependent chromatin remodeling complex that changes the accessibility of transcription factors to DNA. It contains different subunits, including the core ATPase subunit Brg1 or Brm. The SWI/SNF subunits play important roles in CM differentiation and development. During zebrafish heart regeneration, *brg1*, *baf60c*, and *baf180* are induced after injury. Brg1 regulates CM proliferation by interacting with Dnmt3ab to increase methylation of the *cdkn1c* promoter (Xiao et al. 2016). The expression of Baf60c significantly increases during neonatal mouse heart regeneration, and knockdown of *Baf60c* suppresses CM proliferation (Nakamura et al. 2016). These studies suggest that injury-induced expression of the SWI/SNF complex modulates chromatin structure and gene transcription during heart regeneration. Others have reported that histone modification of H3K27me3 is essential for the silencing of structural genes, and conditional over-expression of H3.3K27M mutant variants decreases the K27me3 level and as a result, inhibits cardiac regeneration (Ben-Yair et al. 2019).

### Transcription factors and regeneration enhancers

Some embryonic cardiac transcriptional factors are re-activated after ventricular resection in zebrafish (Lepilina et al. 2006). Transcriptional factors such as Gata4, Hand2, Stat3, and NF-_k_B have essential functions during heart regeneration as reviewed previously (Pronobis and Poss 2020). In the neonatal mouse heart, transcription factors Meis1, Gata4, and Tbx20 play important roles in CM proliferation by regulating the expression of cell-cycle genes. Conditional knockout of Meis1 in mouse CMs drives mature CMs to re-enter the cell cycle, thus prolonging the regeneration window beyond P7, while the overexpression of Meis1 inhibits heart regeneration in the neonatal mouse (Mahmoud et al. 2013). The cardiac transcription factor Gata4 regulates neonatal heart regeneration via the paracrine factor FGF16. Conditional knockout of Gata4 leads to the loss of regenerative ability in neonatal mice, and overexpression of Fgf16 via AAV9 in the Gata4-deficient heart partially rescues cardiac hypertrophy and improves heart function after injury (Yu et al. 2016). In addition, Tbx20 inhibits the expression of p21 while activates YAP, BMP, and Akt signaling, resulting in CM proliferation and improved cardiac function (Xiang et al. 2016). RE1 silencing transcription factor, a transcriptional inhibitor of neuronal genes, promotes CM proliferation via inhibiting p21 expression after injury (Zhang et al. 2017). Together, these studies suggest that embryonic cardiac transcriptional factors are re-deployed for cardiac regeneration after injury.

It remains to be addressed how injury triggers CM proliferation and heart regeneration in model systems such as the zebrafish and neonatal mouse heart. It is logically hypothesized that injury signals may trigger chromatin remodeling (such as an active or repressive state regulated by the SWI/SNF complex or histone modification) and cardiac transcription factors are then recruited to bind regulatory elements (such as promoters and enhancers). Recent global analyses of transcriptome and histone modifications (H3K4me3/H3K27ac) have identified early responsive regenerative enhancers in the zebrafish, killifish, and neonatal mouse heart (Quaife-Ryan et al. 2017; Kang et al. 2016; Wang et al. 2020). Comparative transcriptome profiling has revealed that a number of genes are activated during both heart and fin regeneration, among which *lepb* is strongly induced after injury in zebrafish (Kang et al. 2016). Further analyses have led to identifying the regulatory elements of *lepb*, which consist of a heart-specific enhancer and a fin-specific enhancer that are active in both the zebrafish and neonatal mouse heart and fins/digits. By creating a biotinylatable H3.3 histone variant in CMs, Poss and colleagues have elegantly performed the histone profiling of putative enhancers with H3.3 occupancies during heart regeneration, and have thus isolated several enhancers that are active in the injured heart (Goldman et al. 2017). Their most recent work applying CRISPR-based deletion of enhancers in zebrafish has further demonstrated that some of the regeneration enhancers are responsible for adjacent gene expression including *lepb*, although others may regulate distant gene expression (Thompson et al. 2020). By taking an evolutionary approach from killifish to zebrafish, other investigators have identified 49 putative enhancers during fin regeneration by integrating transcriptome and H3K27ac profiling (Wang et al. 2020). Interestingly, a large fraction of these enhancers is activated in the blastema cells of the regenerating fin, suggesting their potential roles in fin regeneration. They have further found that the killifish *inhba* regeneration enhancer K-IEN and the zebrafish *inhba* enhancer Z-IEN are active in blastema cells, the essential regenerative cells, while human H-IEN is active in non-regenerative cells. Bioinformatics analysis has revealed multiple AP-1 binding sites in these regeneration enhancers, consistent with recent studies on the potential function of AP-1 family members in initiating organ regeneration (Gehrke et al. 2019; Beisaw et al. 2020). Together, the exciting progress on regeneration enhancers, combined with the identification of regenerative transcription factors, will facilitate our understanding of the complex networks regulating heart regeneration (Table 2).

## Perspective on research directions

**Fig. 2:**
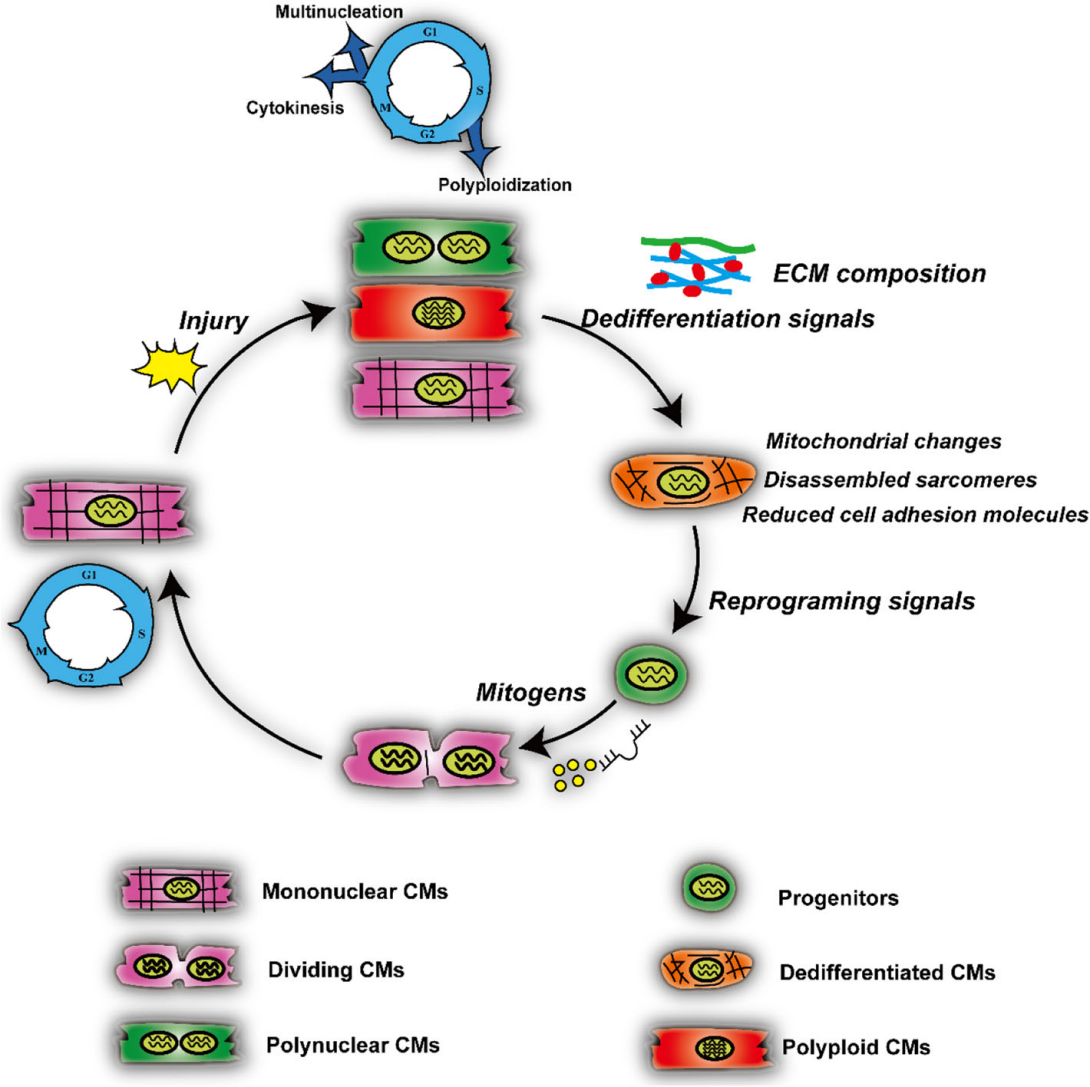
Working model of injury-induced cardiomyocyte proliferation. Injury signals trigger the cell-cycle re-entry of CMs, leading to the formation of mononuclear, polynuclear, or polyploidy CMs. De-differentiation signals together with extracellular matrix (ECM) re-organization then regulate the generation of putative de-differentiated CMs, which have evident changes in mitochondrial morphology, disassembled sarcomeres, and reduced cell adhesion. Finally, reprogramming signals and mitogens drive the formation of putative progenitors and cell division

Inducing CM proliferation has become a promising strategy to develop treatments for heart failure. A large number of studies have shown that cell-cycle regulatory factors, growth factor signaling pathways, miRNAs, transcription factors, and epigenetic modifications stimulate the proliferation of endogenous CMs. In general, injury signals trigger quiescent CMs (mononuclear, polyploid, or multinuclear CMs) into cell-cycle re-entry, via de-differentiation, morphological changes in mitochondria, and reduced cell adhesion, for which reprogramming and mitogenic signals such as Hippo, NRG1/Erbb2/4, FGF, and other signaling pathways are required (Fig. 2). The field has accumulated a mass of data showing that a number of genes and non-coding RNAs are required for CM proliferation, but it remains largely unknown what genetic and epigenetic signals are sufficient to promote CM proliferation and regeneration in non-regenerating hearts such as the adult mammalian heart. To address these unresolved questions, it is necessary to carry out unbiased genetic and/or chemical screens to identify genes and small molecules that promote CM proliferation, such as high-content chemical screens, CRISPR-based genetic screens, and TET-ON based genetic screens (Ma et al. 2017). Recently, a series of studies have succeeded in chemical screening for a variety of biological factors in the mouse and zebrafish (Han et al. 2019; Choi et al. 2013; Magadum et al. 2017). With the rapid development of genomic technology, such as RNA-seq, ATAC-Seq, ChIP-seq, and single-cell sequencing, future studies will continue to decipher the networks that regulate heart regeneration, particularly by investigating the expression and function of putative regenerative enhancers as well as their interactions with transcription factors. Although several genetic and epigenetic factors have been determined to function in CM proliferation, it remains largely unknown how they are coordinated to drive quiescent CMs into cell-cycle re-entry. It has been reported that Hippo/YAP signaling not only interacts with the β-catenin pathway, but also with the PI3K/Akt pathway. Also, NRG1/ErbB2/4 is able to activate both the PI3K/Akt and YAP signaling pathways (Lin et al. 2015; Sudol 2014). Thus, it is very important to address the interplay and mechanisms among the signal pathways that regulate CM proliferation. On the other hand, the current delivery methods for genes and small molecules are mainly systemic delivery, epicardial patches, or direct intramyocardial injection. These delivery methods can also stimulate the proliferation of non-CMs. Therefore, how to improve delivery methods to fine-tune drug release and specifically target CMs awaits further investigation.
